# The genome sequence of the common alder, *Alnus glutinosa* (L.) Gaertn. (Betulaceae)

**DOI:** 10.12688/wellcomeopenres.23137.1

**Published:** 2024-10-07

**Authors:** Maarten J. M. Christenhusz, Zoë Goodwin, David G. Bell, Claudia A. Martin, Royal Botanic Gardens Kew Genome Acquisition Lab, Royal Botanic Garden Edinburgh Genome Acquisition Lab, Darwin Tree of Life Barcoding collective, Plant Genome Sizing collective, Wellcome Sanger Institute Tree of Life Management, Samples and Laboratory team, Wellcome Sanger Institute Scientific Operations: Sequencing Operations, Wellcome Sanger Institute Tree of Life Core Informatics team, Tree of Life Core Informatics collective, Darwin Tree of Life Consortium

**Affiliations:** 1 Royal Botanic Gardens Kew, Richmond, England, UK; 2 Royal Botanic Garden Edinburgh, Edinburgh, Scotland, UK; 3 University of Edinburgh, Edinburgh, Scotland, UK

**Keywords:** Alnus glutinosa, common alder, genome sequence, chromosomal, Fagales

## Abstract

We present a genome assembly of a diploid specimen of *Alnus glutinosa* (the common alder; Streptophyta; Magnoliopsida; Fagales; Betulaceae). The genome sequence has a total length of 456.80 megabases. Most of the assembly is scaffolded into 14 chromosomal pseudomolecules. The mitochondrial genome assemblies have lengths of 505.23 and 155.85 kilobases and the plastid genome is 160.82 kilobases long. Gene annotation of this assembly on Ensembl identified 23,728 protein-coding genes.

## Species taxonomy

Eukaryota; Viridiplantae; Streptophyta; Streptophytina; Embryophyta; Tracheophyta; Euphyllophyta; Spermatophyta; Magnoliopsida; Mesangiospermae; eudicotyledons; Gunneridae; Pentapetalae; rosids; fabids; Fagales; Betulaceae; *Alnus*; *Alnus glutinosa* (L.) Gaertn. (NCBI:txid3517).

## Background

The common alder, *Alnus glutinosa*, also known as black alder or European alder, is a short-lived, deciduous tree to 30 m tall, belonging to the family Betulaceae. Predominantly found in moist environments, this species plays a pivotal role in riparian ecosystems, wet woodlands, along riverbanks, lakeshores and in marshy areas ( Stroh *et al.*, 2023). In some places the species can be dominant where they are the main element of alder carr.

*Alnus glutinosa* is deciduous, and leaves emerge late in spring and drop in autumn. Flowering precedes leaf emergence in spring, with male flowers arranged in catkins that release pollen in the wind, and female catkins united in dense ellipsoid clusters that resemble small cones. Once pollinated by wind, the female catkins become woody, cone-like fruit structures that mature in autumn and persist through winter, gradually releasing seeds that are initially dispersed by wind, but often dispersal is aided by watercourses. The seeds are an important source of food for birds in the winter. The epithet *glutinosa* is Latin for sticky, and refers particularly to young shoots and leaves that are sticky to the touch.

*Alnus glutinosa* is noted for its nitrogen-fixing ability, accomplished through a symbiotic relationship with the actinomycete bacterium *Frankia alni* (Woronin, 1866) Von Tubeuf 1895 ( Paschke *et al.*, 1989; Pawlowski & Newton, 2010) forming nodules on the roots. This relationship enables *A. glutinosa* to thrive in nutrient-poor soils, enhancing soil fertility and promoting plant biodiversity in its habitat, making it a keystone species in wetland ecosystems. It plays a significant role in these ecosystems by stabilising soil and reducing erosion, as well as regulating water levels. The ability of the tree to improve soil fertility through nitrogen fixation makes it beneficial in agroforestry systems and land reclamation projects. *A. glutinosa* can also grow in drier locations and sometimes occurs in mixed woodland.

*Alnus glutinosa* is native to Europe, southwest Asia and northern Africa. Its range extends from Scandinavia and Finland to the Mediterranean and from Ireland to the Caucasus and Volga Valley. It has naturalised in the Azores, Shetland, northeastern North America, southern South America, Tasmania, New Zealand and South Africa ( POWO, 2024). It is widely distributed in the UK and Ireland and thrives in various wetland habitats, with significant populations in bogs, marshes and in the floodplains of all major rivers and along lakeshores ( Preston *et al.*, 2002; Stace *et al.*, 2019; Stroh *et al.*, 2023). Its adaptability to waterlogged soil conditions has facilitated its spread across diverse regions in its native range where many other tree species are unable to cope. In combination with its ability to tolerate periodic droughts, this makes *A. glutinosa* resilient to climate change. The genus *Alnus* has 41 accepted species, some of which have also established populations in Ireland and the UK (e.g. *A. cordata* (Loisel.) Duby, *A. incana* (L.) Moench, *A. rubra* Bong.) *. Alnus glutinosa* is the only native species of the genus in Britain and Ireland ( POWO, 2024).

Some alders in Britain have been infected by a fungal pathogen, *Phytophthora alni* Brasier & S.A.Kirk, sometimes known as alder dieback. This pathogen was first recorded in the UK in 1993, and has become widespread. Evolution of hybrid strains of this fungal pathogen may increase the susceptibility of European alder species to the disease, hence its impact is predicted to increase over time ( Brasier *et al.*, 2004; Fuller *et al.*, 2023). This infection causes the death of roots and patches of bark. Dark spots can also form near the base of the trunk, and the leaves often turn yellow in summer.

Numerous other fungi grow on alder, most of which are mutualistic or benign. Several species only grow in association with *Alnus glutinosa*. *Taphrina alni* (Berk. & Broome) Gjaerum, a fungal pathogen causes alder tongue galls. Another harmless gall is caused by *Eriophyes laevis* (Nalepa, 1889), a midge that sucks sap from leaves, resulting in small pustules on the leaf tissue. While a spider mite, *Aceria nalepai* (Fockeu, 1890), also causes small galls that form mostly on the midveins. Two lichen species are found only (or mostly) on alder: *Stenocybe pullatula* (Ach.) Stein and *Menegazzia terebrata* (Hoffm.) A.Massal. Dozens of insect species are known to feed on alder leaves, including several that are specific to alder, such as the striped alder sawfly ( *Hemichroa crocea* (Geoffroy, 1785)), the May Highflyer ( *Hydriomena impluviata* (Denis & Schiffermüller, 1775)), the Dingy Shell ( *Euchoeca nebulata* (Scopoli, 1763)) and the Alder Kitten moth ( *Furcula bicuspis* (Borkhausen, 1790)). The trees also provide shelter for aquatic animals and for breeding birds. The rare fern *Dryopteris cristata* (L.) A.Gray is associated with alder carr.

Alder has been valued for a variety of ecological and construction uses. Its wood is resistant to water and has been employed in the construction of underwater structures, including bridges, docks and mills. It was often coppiced with the wood used for water pipes or for high quality charcoal made into gunpowder or gas mask filters. Shepherds also carved the wood into flutes and sometimes clogs ( De Cleene & Lejeune, 2000).

*Alnus glutinosa* also has a rich cultural history in European folklore. In Norse mythology, the first woman was carved from alder wood. It was also considered a sacred tree by the Celts, who believed it possessed protective qualities. In the traditional Irish folksong *Song of the Forest Trees*, alder is described as “battle-witch of all woods, tree that is hottest in the fight”. Cutting alder trees in Ireland was once forbidden, but the wood had sometimes been fashioned into warrior shields by Celtic tribes. Because alder wood has a vibrant orange-red inner bark and the white wood turns red after logging, it became associated with blood. This and the fact that alder forms dark thickets in mysterious places like swamps led to its use in various rituals in local cults. It often has negative connotations, ranging from the folklore tale that the red colour resulted from the devil beating his grandmother with alder twigs, to the idea that witches used it to influence the weather. Alder also had positive folklore associated with it, such as fending off devils and witches, and blessing seeds before sowing, so that the birds would leave them alone ( De Cleene & Lejeune, 2000).

Alder leaves and bark have been utilised in traditional medicine for their anti-inflammatory and anti-aging properties. Poultices were commonly made from fresh alder leaves to heal infections, following the medical wisdom from ancient times, but maintained until the 20th century. Bark from young twigs can be brewed into a drink that helps lower fever. This liquid is still used to treat mouth sores, angina and tonsilitis ( De Cleene & Lejeune, 2000).

The common alder has a diploid chromosome number of 2 *n* = 28. Here, we present the first chromosome-level *Alnus glutinosa* genome, which will provide valuable insights into the genetic basis of its ecological adaptations (including host-pathogen evolution), symbiotic relationships and evolutionary history. This genomic resource will be instrumental for conservation efforts, breeding programmes and understanding the role of alder in ecosystem services. Additionally, it holds potential economic and social benefits, such as improving land reclamation strategies and enhancing the ecological health of riparian and wetland habitats.

## Genome sequence report

**Figure 1.**
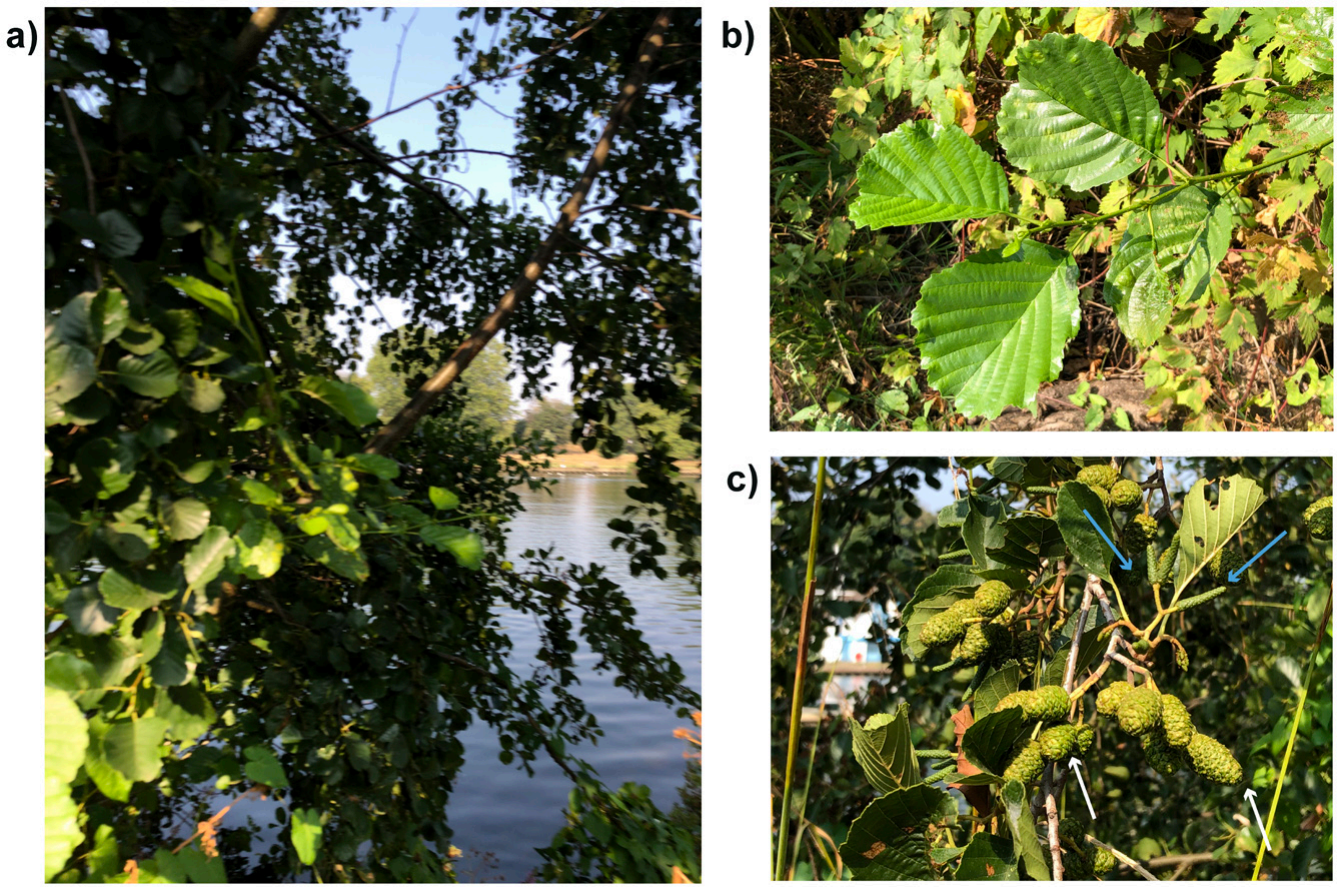
Photographs of the *Alnus glutinosa* (dhAlnGlut1) specimen used for genome sequencing. **a**) Habitat along River Thames in Canbury Gardens. **b**) leaves. **c**) female fruits (white arrows) and young male catkins (blue arrows).

The genome of an *Alnus glutinosa* specimen ( Figure 1) was sequenced, based on a total of 38-fold coverage in Pacific Biosciences single-molecule HiFi long reads and 95-fold coverage in 10X Genomics read clouds. Using flow cytometry, the genome size (1C-value) of the *Alnus glutinosa* specimen was estimated to be 0.67 pg, equivalent to 660 Mb.

Primary assembly contigs were scaffolded with chromosome conformation Hi-C data, which produced 79.17 Gbp from 524.31 million reads, yielding an approximate coverage of 173-fold. Specimen and sequencing information is summarised in Table 1.

**Table 1.** Specimen and sequencing data for *Alnus glutinosa*.

Project information			
**Study title**	Alnus glutinosa		
**Umbrella BioProject**	PRJEB46320		
**Species**	*Alnus glutinosa*		
**BioSample**	SAMEA7522050		
**NCBI taxonomy ID**	3517		
Specimen information			
**Technology**	**ToLID**	**BioSample accession**	**Organism part**
**PacBio long read sequencing**	dhAlnGlut1	SAMEA7522127	leaf
**Hi-C sequencing**	dhAlnGlut1	SAMEA7522125	leaf
**RNA sequencing**	dhAlnGlut2	SAMEA7536056	leaf
Sequencing information			
**Platform**	**Run accession**	**Read count**	**Base count (Gb)**
**Hi-C Illumina NovaSeq 6000**	ERR6688533	5.24e+08	79.17
**PacBio Sequel II**	ERR6808004	7.72e+05	10.32
**PacBio Sequel IIe**	ERR6939242	6.14e+05	8.76
**Chromium Illumina NovaSeq 6000**	ERR6688529	1.06e+08	16.07
**Chromium Illumina NovaSeq 6000**	ERR6688530	1.21e+08	18.32
**Chromium Illumina NovaSeq 6000**	ERR6688531	1.08e+08	16.28
**Chromium Illumina NovaSeq 6000**	ERR6688532	1.11e+08	16.77
**RNA Illumina HiSeq 4000**	ERR9435007	4.47e+07	6.75

**Figure 2.**
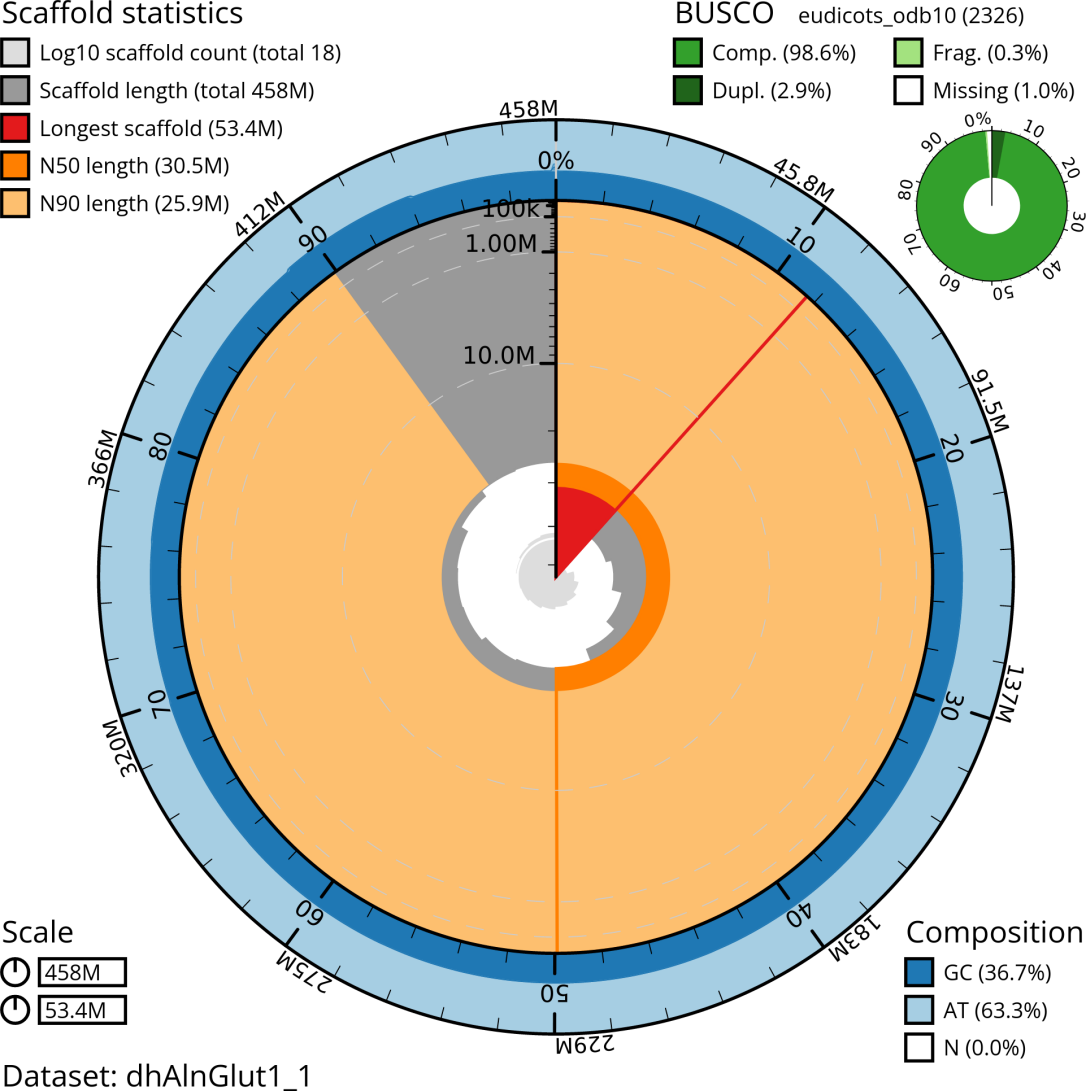
Genome assembly of *Alnus glutinosa*, dhAlnGlut1.1: metrics. The BlobToolKit snail plot shows N50 metrics and BUSCO gene completeness. The main plot is divided into 1,000 size-ordered bins around the circumference with each bin representing 0.1% of the 457,583,206 bp assembly. The distribution of scaffold lengths is shown in dark grey with the plot radius scaled to the longest scaffold present in the assembly (53,352,176 bp, shown in red). . Orange and pale-orange arcs show the N50 and N90 scaffold lengths (30,513,540 and 25,901,623 bp), respectively. The pale grey spiral shows the cumulative scaffold count on a log scale with white scale lines showing successive orders of magnitude. The blue and pale-blue area around the outside of the plot shows the distribution of GC, AT and N percentages in the same bins as the inner plot. A summary of complete, fragmented, duplicated and missing BUSCO genes in the eudicots_odb10 set is shown in the top right. An interactive version of this figure is available at https://blobtoolkit.genomehubs.org/view/dhAlnGlut1_1/dataset/dhAlnGlut1_1/snail.

**Figure 3.**
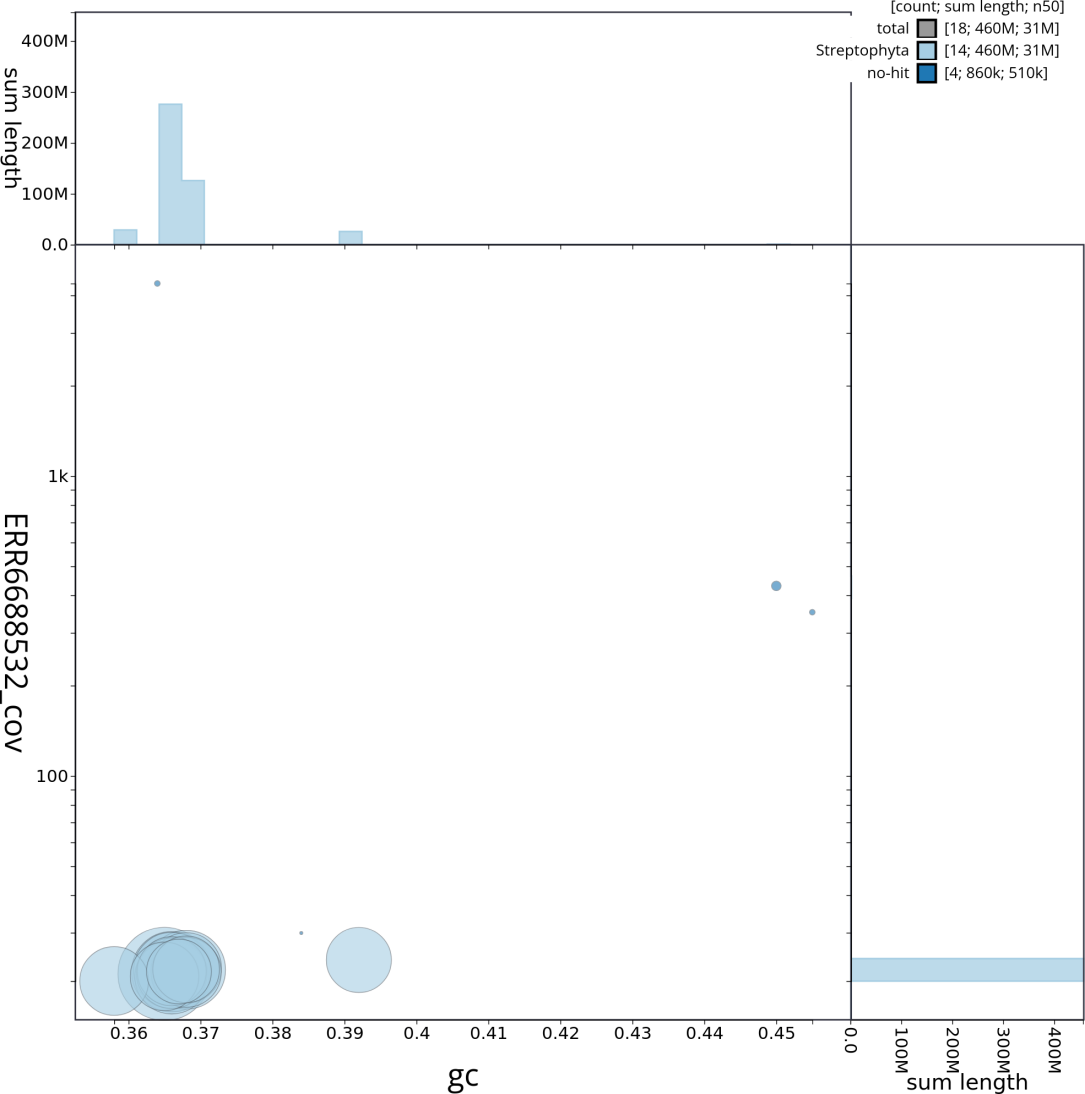
Genome assembly of *Alnus glutinosa*, dhAlnGlut1.1: Blob plot of base coverage against GC proportion for sequences in the assembly. Sequences are coloured by phylum. Circles are sized in proportion to sequence length. Histograms show the distribution of sequence length sum along each axis. An interactive version of this figure is available at https://blobtoolkit.genomehubs.org/view/dhAlnGlut1_1/dataset/dhAlnGlut1_1/blob.

**Figure 4.**
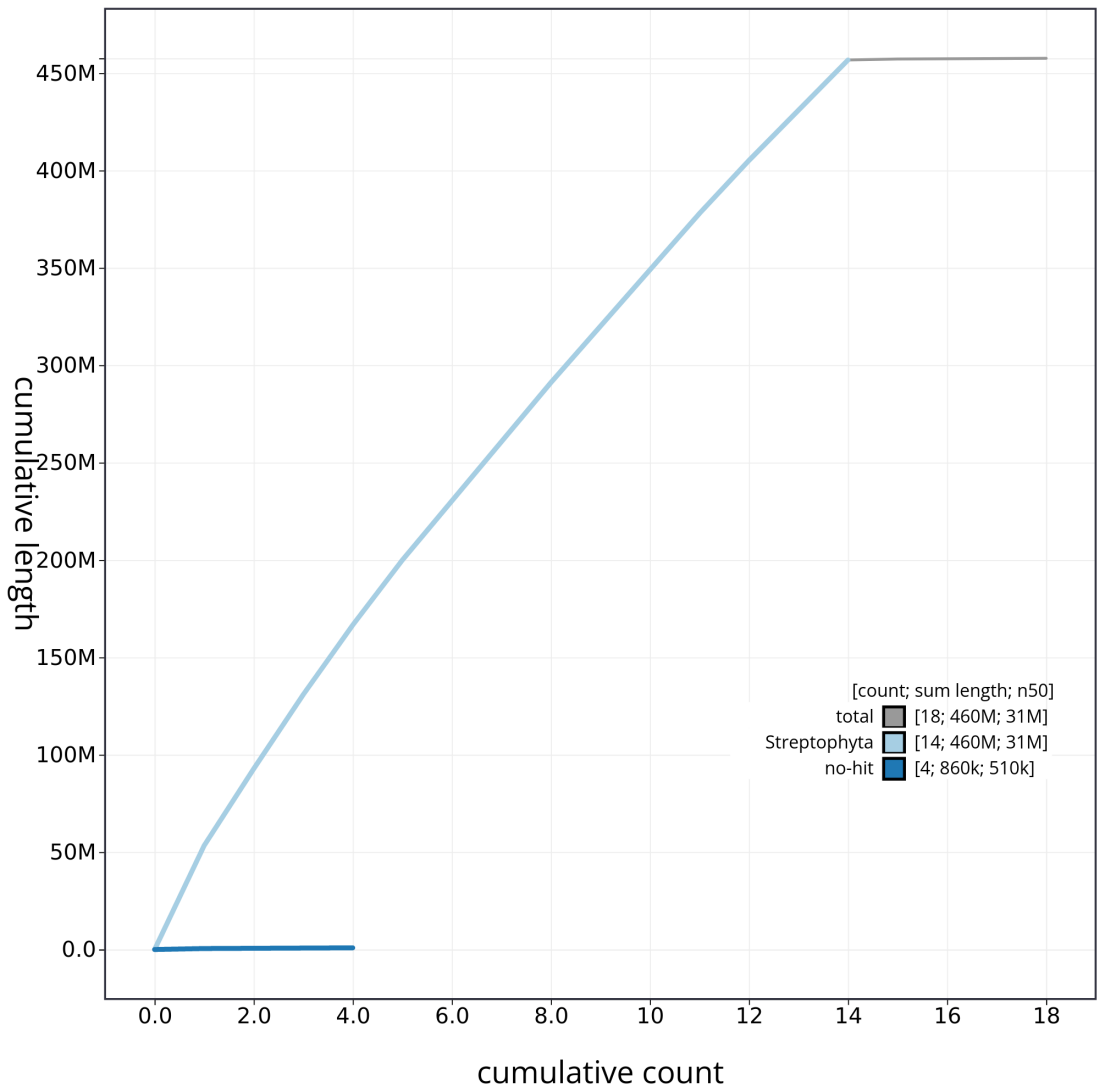
Genome assembly of *Alnus glutinosa* dhAlnGlut1.1: BlobToolKit cumulative sequence plot. The grey line shows cumulative length for all sequences. Coloured lines show cumulative lengths of sequences assigned to each phylum using the buscogenes taxrule. An interactive version of this figure is available at https://blobtoolkit.genomehubs.org/view/dhAlnGlut1_1/dataset/dhAlnGlut1_1/cumulative.

**Figure 5.**
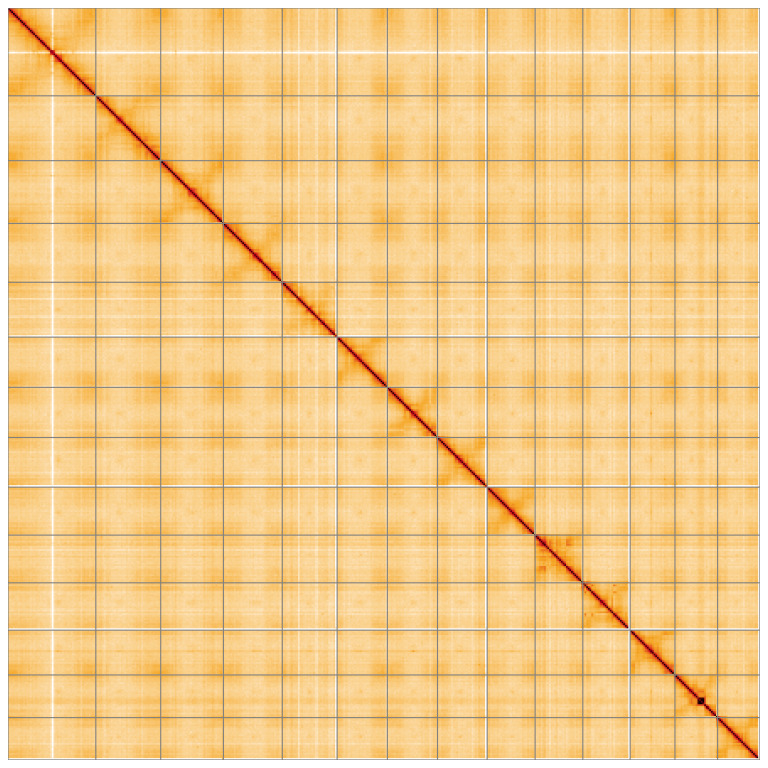
Genome assembly of *Alnus glutinosa*, dhAlnGlut1.1: Hi-C contact map of the dhAlnGlut1.1 assembly, visualised using HiGlass. Chromosomes are shown in order of size from left to right and top to bottom. An interactive version of this figure may be viewed at https://genome-note-higlass.tol.sanger.ac.uk/l/?d=ewauaBn_S4-EMrvmkdS1-g.

Manual assembly curation corrected 87 missing joins or mis-joins, reducing the assembly length by 6.9%. The final assembly has a total length of 456.80 Mb in 15 sequence scaffolds with a scaffold N50 of 30.5 Mb ( Table 2) with 96 gaps. The snail plot in Figure 2 provides a summary of the assembly statistics, while the distribution of assembly scaffolds based on GC proportion and coverage is shown in Figure 3. The cumulative assembly plot in Figure 4 shows curves for subsets of scaffolds assigned to different phyla. Most (99.81%) of the assembly sequence was assigned to 14 chromosomal-level scaffolds. Chromosome-scale scaffolds confirmed by the Hi-C data are named in order of size ( Figure 5; Table 3). On chromosome 12, a region of tandem repeats was observed at 14.75–18.37 Mb. Order and orientation of scaffolds in this region is unknown. While not fully phased, the assembly deposited is of one haplotype. Contigs corresponding to the second haplotype have also been deposited. The mitochondrial and plastid genomes were also assembled and can be found as contigs within the multifasta file of the genome submission.

**Table 2.** Genome assembly data for *Alnus glutinosa*, dhAlnGlut1.1.

Genome assembly		
Assembly name	dhAlnGlut1.1	
Assembly accession	GCA_958979055.1	
*Accession of alternate haplotype*	*GCA_958979045.1*	
Span (Mb)	456.80	
Number of contigs	114	
Contig N50 length (Mb)	24.4	
Number of scaffolds	15	
Scaffold N50 length (Mb)	30.5	
Longest scaffold (Mb)	53.35	
Assembly metrics *		*Benchmark*
Consensus quality (QV)	52.4	*≥ 50*
*k*-mer completeness	99.98%	*≥ 95%*
BUSCO **	C:98.6%[S:95.7%,D:2.9%], F:0.3%,M:1.0%,n:2,326	*C ≥ 95%*
Percentage of assembly mapped to chromosomes	99.81%	*≥ 95%*
Organelles	Mitochondrial genome: 505.23 and 155.85 kb Plastid genome: 160.82 kb	*complete single alleles*
Genome annotation of assembly GCA_958979055.1 at Ensembl		
Number of protein-coding genes	23,728	
Number of non-coding genes	4,860	
Number of gene transcripts	36,650	

* Assembly metric benchmarks are adapted from column VGP-2020 of “Table 1: Proposed standards and metrics for defining genome assembly quality” from Rhie *et al.* (2021).

** BUSCO scores based on the eudicots_odb10 BUSCO set using version 5.4.3. C = complete [S = single copy, D = duplicated], F = fragmented, M = missing, n = number of orthologues in comparison. A full set of BUSCO scores is available at https://blobtoolkit.genomehubs.org/view/dhAlnGlut1_1/dataset/dhAlnGlut1_1/busco.

**Table 3.** Chromosomal pseudomolecules in the genome assembly of *Alnus glutinosa*, dhAlnGlut1.

INSDC accession	Name	Length (Mb)	GC%
OY340898.1	1	53.35	36.5
OY340899.1	2	39.4	36.5
OY340900.1	3	38.11	37.0
OY340901.1	4	35.74	36.5
OY340902.1	5	33.31	36.5
OY340903.1	6	30.51	37.0
OY340904.1	7	30.49	36.5
OY340905.1	8	30.14	37.0
OY340906.1	9	29.14	36.5
OY340907.1	10	28.96	36.0
OY340908.1	11	28.76	36.5
OY340909.1	12	27.21	37.0
OY340910.1	13	25.9	39.0
OY340911.1	14	25.68	36.5
OY340914.1	Pltd	0.16	36.5
OY340912.1	MT1	0.51	45.0
OY340913.1	MT2	0.16	45.5

The estimated Quality Value (QV) of the final assembly is 52.4 with *k*-mer completeness of 99.98%, and the assembly has a BUSCO v5.4.3 completeness of 98.6% (single = 95.7%, duplicated = 2.9%), using the eudicots_odb10 reference set ( *n* = 2,326).

Metadata for specimens, BOLD barcode results, spectra estimates, sequencing runs, contaminants and pre-curation assembly statistics are given at https://links.tol.sanger.ac.uk/species/3517.

## Genome annotation report

The *Alnus glutinosa* genome assembly (GCA_958979055.1) was annotated at the European Bioinformatics Institute (EBI) on Ensembl Rapid Release. The resulting annotation includes 36,650 transcribed mRNAs from 23,728 protein-coding and 4,860 non-coding genes ( Table 2; https://rapid.ensembl.org/Alnus_glutinosa_GCA_958979055.1/Info/Index). The average transcript length is 4,410.79. There are 1.28 coding transcripts per gene and 5.08 exons per transcript.

## Methods

### Sample acquisition, DNA barcoding and genome size estimation

A specimen of *Alnus glutinosa* (specimen ID KDTOL10041, ToLID dhAlnGlut1) was hand-picked from Canbury Gardens, Kingston upon Thames, Surrey, UK (latitude 51.42, longitude –0.31) on 2020-08-12. The specimen was collected and identified by Maarten Christenhusz (Royal Botanic Gardens, Kew, UK) and preserved by freezing at –80 °C. The herbarium voucher associated with the sequenced plant (K001400626) is from the collection number Christenhusz 9038 and is deposited in the herbarium of RBG Kew (K).

The specimen used for RNA sequencing (specimen ID EDTOL00069, ToLID dhAlnGlut2) was collected from Royal Botanic Garden Edinburgh (Inverleith), Midlothian, Scotland, UK (latitude 55.97, longitude –3.21) on 2020-08-12. The specimen was collected by Zoe Goodwin and David Bell and preserved by snap freezing in liquid nitrogen.

The initial species identification was verified by an additional DNA barcoding process according to the framework developed by Twyford *et al*. (2024). A small sample was dissected from each specimen and stored in silica ( Chase & Hills, 1991), while the remaining parts of the specimen were flash frozen at –80°C and shipped on dry ice to the Wellcome Sanger Institute (WSI). The silica-dried tissue was lysed, barcode region(s) amplified by PCR, and amplicons were sequenced and compared to a sequence database ( Crowley *et al.*, 2023). Following whole genome sequence generation, the relevant DNA barcode region was also used alongside the initial barcoding data for sample tracking at the WSI ( Twyford *et al.*, 2024). The standard operating procedures for Darwin Tree of Life barcoding have been deposited in protocols.io ( Beasley *et al.*, 2023).

The genome size was estimated by flow cytometry using the fluorochrome propidium iodide and following the ‘one-step’ method as outlined in Pellicer *et al.* (2021). For this species, the General Purpose Buffer (GPB) supplemented with 3% PVP and 0.08% (v/v) beta-mercaptoethanol was used for isolation of nuclei ( Loureiro *et al.*, 2007), and the internal calibration standard was *Petroselinum crispum* ‘Champion Moss Curled’ with an assumed 1C-value of 2,200 Mb ( Obermayer *et al.*, 2002).

### Nucleic acid extraction

The workflow for high molecular weight (HMW) DNA extraction at the WSI Tree of Life Core Laboratory includes a sequence of core procedures: sample preparation and homogenisation, DNA extraction, fragmentation and purification. Detailed protocols are available on protocols.io ( Denton *et al.*, 2023). The dhAlnGlut1 sample was weighed and dissected on dry ice ( Jay *et al.*, 2023), and leaf tissue was cryogenically disrupted using the Covaris cryoPREP ^®^ Automated Dry Pulverizer ( Narváez-Gómez *et al.*, 2023). HMW DNA was extracted using the Automated Plant MagAttract v1 protocol ( Sheerin *et al.*, 2023). HMW DNA was sheared into an average fragment size of 12–20 kb in a Megaruptor 3 system ( Todorovic *et al.*, 2023). Sheared DNA was purified by solid-phase reversible immobilisation, using AMPure PB beads to eliminate shorter fragments and concentrate the DNA ( Strickland *et al.*, 2023). The concentration of the sheared and purified DNA was assessed using a Nanodrop spectrophotometer and Qubit Fluorometer and Qubit dsDNA High Sensitivity Assay kit. Fragment size distribution was evaluated by running the sample on the FemtoPulse system.

RNA was extracted from leaf tissue of dhAlnGlut2 in the Tree of Life Laboratory at the WSI using the RNA Extraction: Automated MagMax™ *mir*Vana protocol ( do Amaral *et al.*, 2023). The RNA concentration was assessed using a Nanodrop spectrophotometer and a Qubit Fluorometer using the Qubit RNA Broad-Range Assay kit. Analysis of the integrity of the RNA was done using the Agilent RNA 6000 Pico Kit and Eukaryotic Total RNA assay.

### Sequencing

Table 1 gives the raw read accessions and read and base counts for each sequencing technology.

Legacy Chromium10X Genomics read cloud DNA sequencing libraries were constructed according to the manufacturers’ instructions and sequencing was performed on the Illumina NovaSeq 6000 instrument.

Pacific Biosciences HiFi circular consensus DNA sequencing libraries were constructed according to the manufacturers’ instructions. Poly(A) RNA-Seq libraries were constructed using the NEB Ultra II RNA Library Prep kit. DNA and RNA sequencing was performed by the Scientific Operations core at the WSI on Pacific Biosciences Sequel IIe (HiFi) and Illumina HiSeq 4000 (RNA-Seq) instruments.

Hi-C data were generated from the leaf tissue of dhAlnGlut1, using the Arima-HiC v2 kit. In brief, frozen tissue (–80°C) was fixed, and the DNA crosslinked using a TC buffer containing formaldehyde. The crosslinked DNA was then digested using a restriction enzyme master mix. The 5’-overhangs were then filled in and labelled with a biotinylated nucleotide and proximally ligated. The biotinylated DNA construct was fragmented to a fragment size of 400 to 600 bp using a Covaris E220 sonicator. The DNA was then enriched, barcoded, and amplified using the NEBNext Ultra II DNA Library Prep Kit, following manufacturers’ instructions. The Hi-C sequencing was performed using paired-end sequencing with a read length of 150 bp on an Illumina NovaSeq 6000 instrument.

### Genome assembly, curation and evaluation

***Assembly***

The HiFi reads were first assembled using Hifiasm ( Cheng *et al.*, 2021) with the --primary option. One round of polishing was performed by aligning 10X Genomics read data to the assembly with Long Ranger ALIGN, calling variants with FreeBayes ( Garrison & Marth, 2012). Haplotypic duplications were identified and removed with purge_dups ( Guan *et al.*, 2020). The assembly was then scaffolded with Hi-C data ( Rao *et al.*, 2014) using SALSA2 ( Ghurye *et al.*, 2019). The scaffolded assemblies were evaluated using Gfastats ( Formenti *et al.*, 2022), BUSCO ( Manni *et al.*, 2021) and MERQURY.FK ( Rhie *et al.*, 2020). The organelle genomes were assembled using OATK ( Zhou, 2023).

***Curation***

The assembly was checked for contamination and corrected using the gEVAL system ( Chow *et al.*, 2016) as described previously ( Howe *et al.*, 2021). Manual curation was performed using gEVAL, HiGlass ( Kerpedjiev *et al.*, 2018) and Pretext ( Harry, 2022). Scaffolds were visually inspected and any identified contamination, missed joins, and mis-joins were corrected, and duplicate sequences were tagged and removed. The curation process is documented at https://gitlab.com/wtsi-grit/rapid-curation (article in preparation).

***Evaluation of final assembly***

A Hi-C map for the final assembly was produced using bwa-mem2 ( Vasimuddin *et al.*, 2019) in the Cooler file format ( Abdennur & Mirny, 2020). To assess the assembly metrics, the *k*-mer completeness and QV consensus quality values were calculated in Merqury ( Rhie *et al.*, 2020). This work was done using the “sanger-tol/readmapping” ( Surana *et al.*, 2023a) and “sanger-tol/genomenote” ( Surana *et al.*, 2023b) pipelines. The genome readmapping pipelines were developed using the nf-core tooling ( Ewels *et al.*, 2020), use MultiQC ( Ewels *et al.*, 2016), and make extensive use of the Conda package manager, the Bioconda initiative ( Grüning *et al.*, 2018), the Biocontainers infrastructure ( da Veiga Leprevost *et al.*, 2017), and the Docker ( Merkel, 2014) and Singularity ( Kurtzer *et al.*, 2017) containerisation solutions. The genome was analysed within the BlobToolKit environment ( Challis *et al.*, 2020) and BUSCO scores ( Manni *et al.*, 2021) were calculated.

Table 4 contains a list of relevant software tool versions and sources.

**Table 4.** Software tools: versions and sources.

Software tool	Version	Source
BlobToolKit	4.1.7	https://github.com/blobtoolkit/blobtoolkit
BUSCO	5.3.2	https://gitlab.com/ezlab/busco
FreeBayes	1.3.1-17- gaa2ace8	https://github.com/freebayes/freebayes
gEVAL	N/A	https://geval.org.uk/
Hifiasm	0.15.3	https://github.com/chhylp123/hifiasm
HiGlass	1.11.6	https://github.com/higlass/higlass
Long Ranger ALIGN	2.2.2	https://support.10xgenomics.com/genome-exome/software/pipelines/ latest/advanced/other-pipelines
Merqury	MerquryFK	https://github.com/thegenemyers/MERQURY.FK
OATK	0.2	https://github.com/c-zhou/oatk
PretextView	0.2	https://github.com/wtsi-hpag/PretextView
purge_dups	1.2.3	https://github.com/dfguan/purge_dups
SALSA	2.2	https://github.com/salsa-rs/salsa
sanger-tol/ genomenote	v1.0	https://github.com/sanger-tol/genomenote
sanger-tol/ readmapping	1.1.0	https://github.com/sanger-tol/readmapping/tree/1.1.0
YaHS	yahs- 1.1.91eebc2	https://github.com/c-zhou/yahs

### Genome annotation

The Ensembl Genebuild annotation system ( Aken *et al.*, 2016) was used to generate annotation for the *Alnus glutinosa* assembly (GCA_958979055.1) in Ensembl Rapid Release at the EBI. Annotation was created primarily through alignment of transcriptomic data to the genome, with gap filling via protein-to-genome alignments of a select set of proteins from UniProt ( UniProt Consortium, 2019).

### Wellcome Sanger Institute – Legal and Governance

The materials that have contributed to this genome note have been supplied by a Darwin Tree of Life Partner. The submission of materials by a Darwin Tree of Life Partner is subject to the **‘Darwin Tree of Life Project Sampling Code of Practice’**, which can be found in full on the Darwin Tree of Life website here. By agreeing with and signing up to the Sampling Code of Practice, the Darwin Tree of Life Partner agrees they will meet the legal and ethical requirements and standards set out within this document in respect of all samples acquired for, and supplied to, the Darwin Tree of Life Project.

Further, the Wellcome Sanger Institute employs a process whereby due diligence is carried out proportionate to the nature of the materials themselves, and the circumstances under which they have been/are to be collected and provided for use. The purpose of this is to address and mitigate any potential legal and/or ethical implications of receipt and use of the materials as part of the research project, and to ensure that in doing so we align with best practice wherever possible. The overarching areas of consideration are:

•     Ethical review of provenance and sourcing of the material

•     Legality of collection, transfer and use (national and international)

Each transfer of samples is further undertaken according to a Research Collaboration Agreement or Material Transfer Agreement entered into by the Darwin Tree of Life Partner, Genome Research Limited (operating as the Wellcome Sanger Institute), and in some circumstances other Darwin Tree of Life collaborators.

## Data Availability

European Nucleotide Archive: *Alnus glutinosa*. Accession number PRJEB46320; https://identifiers.org/ena.embl/PRJEB46320 ( Wellcome Sanger Institute, 2023). The genome sequence is released openly for reuse. The *Alnus glutinosa* genome sequencing initiative is part of the Darwin Tree of Life (DToL) project. All raw sequence data and the assembly have been deposited in INSDC databases. The genome will be annotated using available RNA-Seq data and presented through the Ensembl pipeline at the European Bioinformatics Institute. Raw data and assembly accession identifiers are reported in Table 1.

## Author information

Members of the Royal Botanic Gardens Kew Genome Acquisition Lab are listed here: https://doi.org/10.5281/zenodo.12625079.

Members of the Royal Botanic Garden Edinburgh Genome Acquisition Lab are listed here: https://doi.org/10.5281/zenodo.4786682.

Members of the Plant Genome Sizing collective are listed here: https://doi.org/10.5281/zenodo.7994306.

Members of the Darwin Tree of Life Barcoding collective are listed here: https://doi.org/10.5281/zenodo.12158331.

Members of the Wellcome Sanger Institute Tree of Life Management, Samples and Laboratory team are listed here: https://doi.org/10.5281/zenodo.12162482.

Members of Wellcome Sanger Institute Scientific Operations: Sequencing Operations are listed here: https://doi.org/10.5281/zenodo.12165051.

Members of the Wellcome Sanger Institute Tree of Life Core Informatics team are listed here: https://doi.org/10.5281/zenodo.12160324.

Members of the Tree of Life Core Informatics collective are listed here: https://doi.org/10.5281/zenodo.12205391.

Members of the Darwin Tree of Life Consortium are listed here: https://doi.org/10.5281/zenodo.4783558.
