# Supplementary material for: The genome sequence of the common alder, Alnus glutinosa (L.) Gaertn. (Betulaceae)
Source: Wellcome Open Res. 2024 Oct 7;9:570. [Version 1] doi: 10.12688/wellcomeopenres.23137.1 (PMC11541074; doi:10.12688/wellcomeopenres.23137.1)
Supplement: Peer review history

# Reviewer report: Reviewer response for version 1

**DOI:** 10.21956/wellcomeopenres.25477.r108087

**Published:** 2024-11-06

**Authors:** Margaret E Staton

**Affiliations:** 1 The University of Tennessee, Knoxville, Tennessee, USA

The authors present a haploid, nonphased reference genome and gene annotation for Alnus glutinosa. It is assembled and annotated using modern methods and appears to be very complete based on kmers, BUSCOs and N50/N90 stats. The only concern I see is that flow cytometry indicates the genome should be 660Mb but the assembly is only 456Mb. Author speculation on this difference would be a nice addition.

Are sufficient details of methods and materials provided to allow replication by others?

Yes

Is the rationale for creating the dataset(s) clearly described?

Yes

Are the datasets clearly presented in a useable and accessible format?

Yes

Are the protocols appropriate and is the work technically sound?

Yes

Reviewer Expertise:

Plant Computational Genomics

I confirm that I have read this submission and believe that I have an appropriate level of expertise to confirm that it is of an acceptable scientific standard.

# Reviewer report: Reviewer response for version 1

**DOI:** 10.21956/wellcomeopenres.25477.r108089

**Published:** 2024-11-05

**Authors:** Parimalan Rangan

**Affiliations:** 1 ICAR-National Bureau of Plant Genetic Resources, New Delhi, India

1. The authors can use other assembly metrics like LAI or GCI to assess the quality of their finished assembly.

 2. HiC contact map may be partitioned along with the chromosomes for easy visualization.

 3. The genes might be subjected with functional annotation so that these can be used to derive out the biological meaning.  Say, for example the genes associated in the symbiotic nitrogen-fixation using an actinomycete Frankia nodulation mechanism.

Are sufficient details of methods and materials provided to allow replication by others?

Yes

Is the rationale for creating the dataset(s) clearly described?

Yes

Are the datasets clearly presented in a useable and accessible format?

Yes

Are the protocols appropriate and is the work technically sound?

Yes

Reviewer Expertise:

Transcriptomics, Genomics, molecular biology, gene cloning

I confirm that I have read this submission and believe that I have an appropriate level of expertise to confirm that it is of an acceptable scientific standard.
